# Analysing the rice young panicle transcriptome reveals the gene regulatory network controlled by *TRIANGULAR HULL1*

**DOI:** 10.1186/s12284-019-0265-2

**Published:** 2019-02-06

**Authors:** Jun Wang, Qiang Zhang, Yi Wang, Jing Huang, Nengjie Luo, Shengbo Wei, Jian Jin

**Affiliations:** 1 State Key Laboratory for Conservation and Utilization of Subtropical Agro-bioresources, Guangxi University, Nanning, 530004 People’s Republic of China; 2 College of Life Science and Technology, Guangxi University, Nanning, 530004 People’s Republic of China; 3 College of Forestry, Guangxi University, Nanning, 530004 People’s Republic of China

**Keywords:** Rice, *TH1*, RNA-seq, Lemma/Palea development, Grain filling

## Abstract

**Background:**

*TRIANGULAR HULL1* (*TH1*), a member of the rice ALOG gene family, has been characterized as a rice lemma/palea-related gene. To understand the gene regulatory network that controlled by *TH1*, we analyzed the transcriptome from a *TH1* knock out (KO) line, which was generated by CRISPR/Cas9. Our study may shed some light on the molecular mechanism of lemma/palea development.

**Results:**

We obtained 20 T_0_ *th1*-C transgenic plants by CRISPR/Cas9. Among the 20 plants, there were eight bi-allelic mutations, five homozygous mutations, three heterozygous mutations, and four Non-KO plants. By comparing with the wild type and the heterozygous knock out (KO) line, the homozygous KO lines showed defects in lemma/palea development as well as in grain filling. Further more, we studied the gene regulatory network that controlled by *TH1* by comparing the transcriptome of a homozygous *TH1* KO line with its Non-KO line as a control. A total of 622 genes were identified as differentially expressed genes (DEGs), of which 297 genes were significantly up-regulated while 325 genes were down-regulated. One hundred thirty eight of the DEGs were assigned to the 59 KEGG (Kyoto Encyclopedia of Genes and Genomes) pathways. Among these annotated DEGs, 15 genes were related to plant hormone signal transduction, eight genes were related to starch and sucrose metabolism. These were the two largest groups of DEGs according to the KEGG pathway analysis.

**Conclusions:**

Our results indicated that hormone related genes and starch/sucrose metabolism related genes might act as downstream targets of *TH1*; they might be responsible for lemma/palea development and grain filling respectively.

**Electronic supplementary material:**

The online version of this article (10.1186/s12284-019-0265-2) contains supplementary material, which is available to authorized users.

## Background

Rice (*Oryza sativa* L.) is one of the most important crops, feeding more than one third of the whole population of the world. The spikelet is a critical unit for rice yield. In rice, one spikelet contains a fertile floret, which consists by one lemma, one palea, two lodicules, six stamens and one pistil. The shape of the grain is largely determined by the development of the lemma and palea. In recent years, a number of genes involved in lemma and palea development have been identified.

*DROOPING LEAF* (*DL*), a member of the *YABBY* gene family, is specifically expressed in the lemma, but not in the palea, it involves in leaf midrib formation and carpel specification. Although its expression is restricted to the lemma, its function on lemma development remains unknown (Yamaguchi et al. 2004; Ishikawa et al. 2009); *MOSAIC FLORAL ORGANS1* (*MFO1*)/*MADS6* is a member of MADS-box gene family that specifies palea identity. In the *mfo1* mutants, the margin of the palea shows an abnormal outgrowth accompanied by an ectopic expression of *DL* (Ohmori et al. 2009; Li et al. 2010); *RETARDED PALEA1* (*REP1*), a TCP-domain protein, is expressed specifically at an early stage in the palea primordial, and later disperses to the lemma, palea and stamen. Transgenic plants overexpressing *REP1* show a similar phenotype to that of *mfo1* (Luo et al. 2005; Yuan et al. 2009); *OsMADS1*/*LHS1* and *OsMADS15* play a critical role in lemma and palea development, loss of function in these two genes causes defects in both lemma and palea development (Jeon et al. 2000; Wang et al. 2010). *PEN BEAK* (*OPB*)/*STAMENLESS1* (*SL1*)/*JAG*/ and *DEGENERATED HULL1* (*DH1*) are also required for normal lemma/palea development (Horigome et al. 2009; Xiao et al. 2009; Duan et al. 2010; Li et al. 2008). Recently research showed that genes involved in the small RNA pathway including *SHOOT ORGANIZATION1* (*SHO1*), *SHOOTLESS2* (*SHL2*), *SHO2*/*SHOOTLESS4* (*SHL4*) and *WAVY LEAF1* (*WAF1*) participate in determining the polarity of the lemma and palea (Itoh et al. 2000; Nagasaki et al. 2007; Abe et al. 2010) .

*TRIANGULAR HULL1* (*TH1*), also known as *BEAK LIKE SPIKELET1* (*BLS1*)/*BEAK-SHAPED GRAIN1* (*BSG1*)/*BEAK-SHAPED HULL1* (*BH1*)/*ABNORMAL FLOWER AND DWARF1* (*AFD1*), is a member of the rice ALOG gene family, it encodes a DUF640 domain transcription factor that associated with grain shape (Iyer and Aravind 2011), and it is expressed mainly in the young panicle and elongating stem. In comparison with the wild type plant, *th1* mutant shows an abnormal beak-shaped grain whose lemma and palea develop incompletely, resulting in the reduction for grain width, and therefore for the yield (Li et al. 2012; Ma et al. 2013; Yan et al. 2013; Wei et al. 2013; Ren et al. 2016). Although *TH1* has been proven to play an important role in lemma/palea development, the regulatory network of *TH1* still remains largely unknown. In the previous research, the mutant lines using for *TH1* characterization were with amino acid change (*bsg1–2*) (Yan et al. 2013), a frame shift that distant from the translation initiation site (*th1–1*, *bsg1–1* and *afd1*) (Li et al. 2012; Yan et al. 2013; Ren et al. 2016) or large fragment deletion (~ 50 kb deletion for *bls1* and ~ 55 kb deletion for *bh1*) (Ma et al. 2013; Wei et al. 2013). In this study, we developed a *TH1* KO line by CRISPR/Cas9 and explored the transcriptional regulatory network controlled by *TH1*.

## Result

### *TH1* KO line was generated using CRISPR/Cas9

**Fig. 1:**
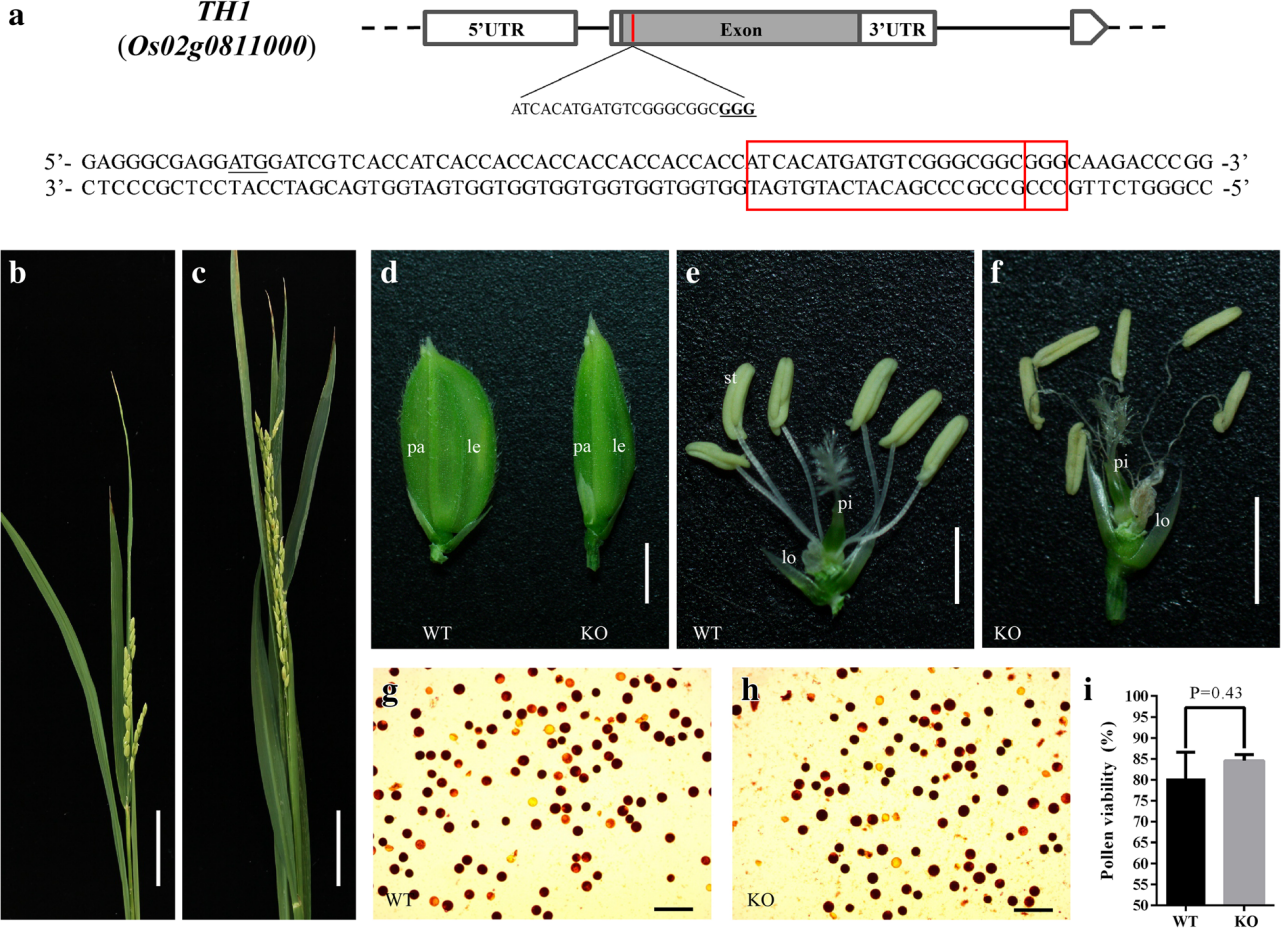
The CRISPR/Cas9 target site of *TH1* and phenotype of the KO plants. **a** Schematic of *TH1* gene structure and the CRISPR/Cas9 target site. *TH1* contains a single exon indicated by gray rectangles. The translation initiation codon (ATG) and termination codon (TGA) are shown. The target site nucleotides are shown in capital letters, and the protospacer adjacent motif (PAM) site is underlined. **b, c** Panicles of wild type and KO plant. **d-f** spikelet of wild type and KO plant. **g, h** Pollen viability test for wild type and KO plant, represents the pollen activity for 3 biological replicates of each line respectively. **i** Statistical data of pollen activity. Values are shown as means ± s.d. (*n* = 3). *P* values were calculated by the Student’s t-test. Scale bar is 5 cm in (**b**) and (**c**), 2 mm in (**d**), **e** and (**f**), 200 μm in (**g**) and (**h**)

To investigate the biological function of *TH1*, we generated a *TH1* KO line *th1*-C by CRISPR/Cas9, with the target site located within the gene at approximately 55 bp downstream of the translation initiation site (Fig. 1a). We obtained 20 T_0_ *th1*-C transgenic plants and analyzed the target site in all the plants (Additional file 1: Table S1). Direct Sanger-sequencing of the target-containing amplicons followed by decoding via DsDecodeM (Liu et al. 2015) showed that among the 20 plants, there were eight bi-allelic mutations, five homozygous mutations, three heterozygous mutations, and four Non-KO plants. Based on allelic mutation types, 15.0% (6/40) of the mutations were nucleotide deletions, while 57.5% (23/40) of the mutations were nucleotide insertions, all of which were 1 bp single insertion (Table 1).

**Table 1:** Ratios of mutant genotype and mutation type at the target site in T_0_ transgenic plants

Mutant genotype ratios (%) ^a^				Mutation type ratios (%) ^b^		
Non-KO	Bi-allele	Homozygote	Heterozygote	Non-KO	Insertion	Deletion
20.0(4/20)	40.0 (8/20)	25.0 (5/20)	15.0 (3/20)	27.5 (11/40)	57.5 (23/40)	15.0 (6/40)

^a^Based on the number of each mutant genotype out of the total number of all mutant genotypes at the target site

^b^Based on the number of each allele mutation type out of the total number of all allele mutation types at the target site

### Homozygous *TH1* KO lines showed defects in lemma/Palea development

During the vegetative growth stage, no phenotypic change could be observed in the T_0_ plants of *th1*-C. Whereas during the reproductive growth stage, by comparing with the wild type and the heterozygous KO line, the homozygous KO lines showed a slender lemma/palea, which displayed a beak-like structure. In addition, unlike those of the wild type plants, the filaments of the KO plants were curled (Fig. 1b-f). In the maturing stage, the homozygous KO lines had a defect in grain filling and were completely infertile. By iodine potassium iodide staining, the pollen activity between homozygous KO line and wild type plant showed no significant difference (Fig. 1g-i). This result indicated that the sterility of the homozygous KO plant might not relate to pollen activity. We also generated a *TH1* overexpression transgenic line, however, no phenotypic change could be observed during the whole developmental stages when compared with the wild type (data not show).

### T_1_ homozygous KO plants were identified for the subsequent RNA-seq

**Fig. 2:**
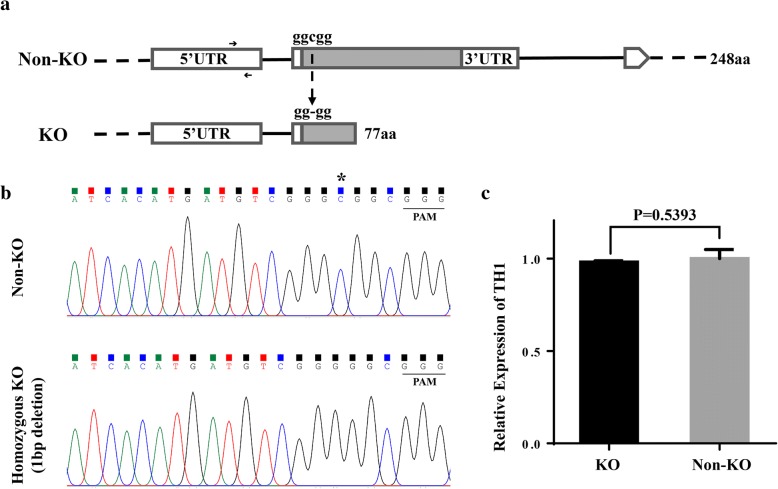
Comparation of *TH1* locus between KO and Non-KO lines. **a** Genomic structures at the *TH1* locus in KO and Non-KO line. Arrows indicate the qRT-PCR primers for *TH1*. **b** The original sequencing chromatograms of the target site for *TH1* in Non-KO and homozygous KO line, which were derived from a T_0_ heterozygous KO plant. The protospacer adjacent motif (PAM) site is underlined, the “C” marked with an asterisk was the deleted in the KO plant. **c** Expression level of *TH1* in KO and Non-KO line. Values are shown as means ± s.d. (*n* = 3). P values were calculated by the Student’s t-test

In order to study the gene regulatory network that controlled by *TH1*, we analyzed the transcriptome data generated from the young panicle (~ 2 cm) of homozygous *th1*-C and Non-KO control plants. Since the T_0_ generation of homozygous KO plants were infertile, T_1_ homozygous KO plants (with 1 bp deletion) and Non-KO plants that derived from a T_0_ heterozygous KO plant (*th1*-C-15) were used for the RNA-seq experiment. By comparing with the Non-KO plant, the homozygous KO plant with 1 bp deletion in the target site leads to a frame shift with premature transcription termination for *TH1* (Fig. 2a-b). By qRT-PCR, we detected no significant difference for *TH1* expression level between KO and Non-KO plants (Fig. 2c).

### Transcriptome analysis

**Fig. 3:**
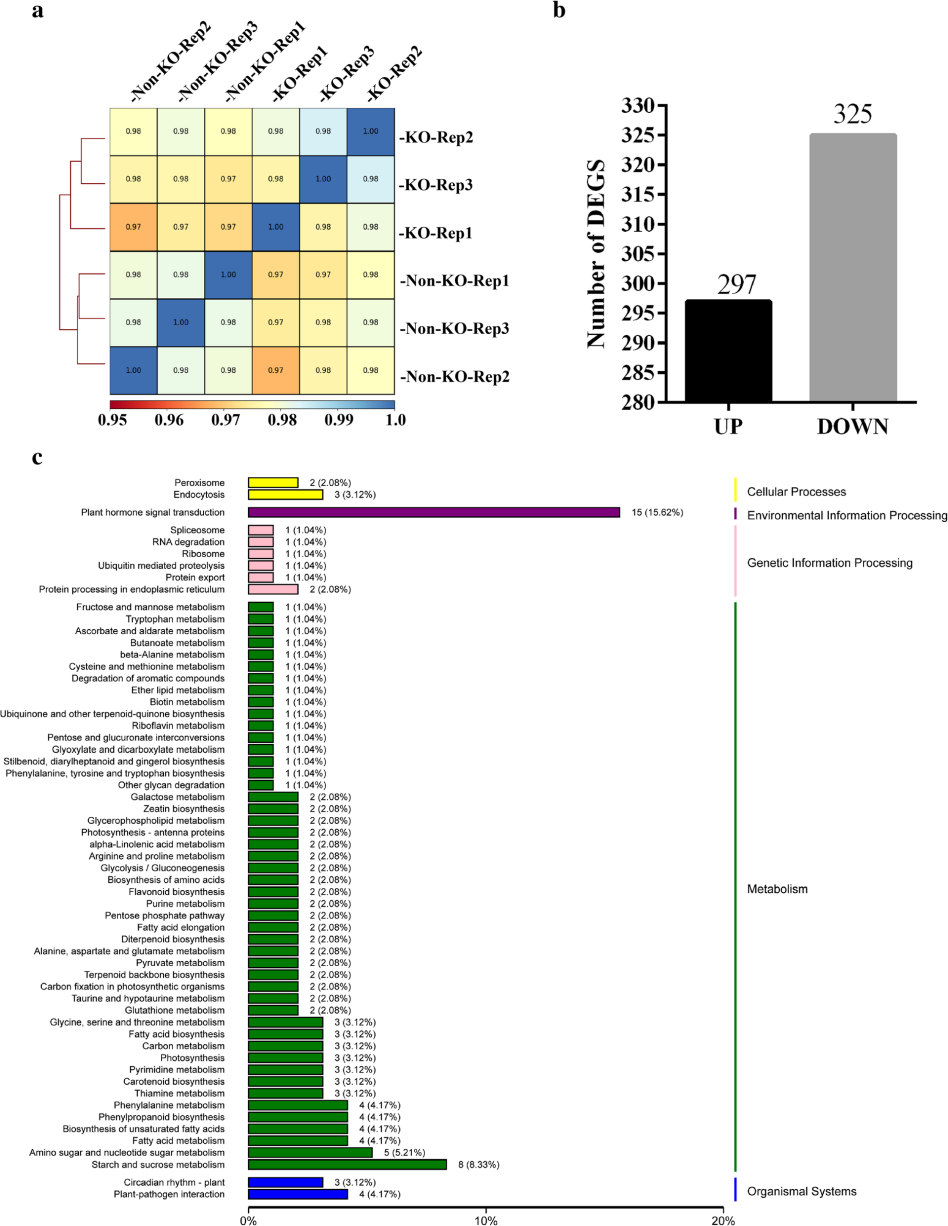
Overview of the DEGs. **a** Correlation between any two replicates was calculated by Spearman Correlation Coefficient value. **b** Numbers of up-regulated and down-regulated DEGs. **c** KEGG pathway assignment of the assembled unigenes (top 50 pathways according to enrichment factor). The vertical axis represents the enriched KEGG pathways and the horizontal axis represents the number of unigenes in each pathway

We carried out the RNA-seq with three biological replicates for KO and Non-KO samples respectively. After filtering, the total number of clean reads per library was 41.16, 46.05 and 44.08 million for KO while 50.45, 43.21 and 45.24 million for Non-KO. For each library, at least 91.82% of the clean reads had a quality score of Q30. The GC content was 51.75%, 51.82% and 52.15% for KO while 51.35%, 51.22% and 51.66% for Non-KO. More than 83.53% of the clean reads were successfully and uniquely mapped to the rice genome using the TopHat2 software (Table 2) (Kim et al. 2013). Additionally, Spearman correlation coefficients between any two replicates were calculated using deepTool2 (Ramírez et al. 2016). The correlation was higher within groups than that between KO and Non-KO samples (Fig. 3a). Taken together, these results indicated the good quality of the sequencing data, suggesting the reliability for subsequent transcriptome analysis results.

**Table 2:** Statistical analysis of transcriptome sequencing data

Sample	Total Reads	Mapped Reads	Mapped ratio	Uniq Mapped Reads	Uniq Mapped ratio	GC Content	% ≥ Q30
KO_REP1	44,162,756	37,777,354	85.54%	36,887,025	83.53%	51.57%	91.82%
KO_REP2	46,049,062	40,954,889	88.94%	39,907,326	86.66%	51.82%	91.90%
KO_REP3	44,084,330	39,067,649	88.62%	38,138,669	86.51%	52.15%	91.86%
Non-KO_REP1	50,452,122	45,162,267	89.52%	44,143,020	87.49%	51.35%	92.15%
Non-KO_REP2	43,214,520	37,838,695	87.56%	36,569,622	84.62%	51.22%	91.92%
Non-KO_REP3	45,241,590	40,278,131	89.03%	39,296,538	86.86%	51.66%	92.10%

Twenty-two thousand seven hundred ninety-seven genes expression level could be detected by RNA-seq at least in one group of the samples (Additional file 2: Table S2). Among these genes, a total of 622 genes were identified as differentially expressed genes (DEGs) with the cut-off for FDR (False Discovery Rate) < 0.05 and FC (Fold Change) ≥ 2. Of these DEGs, 297 genes were significantly up-regulated while 325 genes were down-regulated (Fig. 3b; Additional file 3: Table S3). One hundred fifty DEGs were annotated by KEGG (Kyoto Encyclopedia of Genes and Genomes) (Additional file 4: Table S4). One hundred thirty-eight of 150 DEGs were assigned to the 59 KEGG pathways (Additional file 5: Table S5). Among these KEGG pathway annotated DEGs, 15 genes were related to plant hormone signal transduction, and eight genes were related to starch and sucrose metabolism. These were the two largest groups of DEGs according to the KEGG pathway analysis (Fig. 3c). To further validate the expression profiles of genes in our RNA-seq analyses, 15 genes were selected for expression quantification using qRT-PCR, including five that had been previously characterized as lemma/palea-related genes but did not show differential expression in our experiment (*WAF1*, *OsMADS15*, *SHO1*, *MFO1* and *LHS*) and 10 DEGs that were mainly related to plant hormone signal transduction (*RERJ1*, *Os06g0142400*, *OsJAZ12*, *OsJAZ11*, *OsJAZ10*, *OsJAZ8*, *OsJAZ7*, *OsEIL2*, *OsSAUR23* and *OsSAUR20*). The results revealed that the expression pattern of the selected genes as determined by qRT-PCR were largely identical to those determined by RNA-seq (Additional file 6: Figure S1).

## Discussion

### *TH1* is critical for the rice fertility

**Fig. 4:**
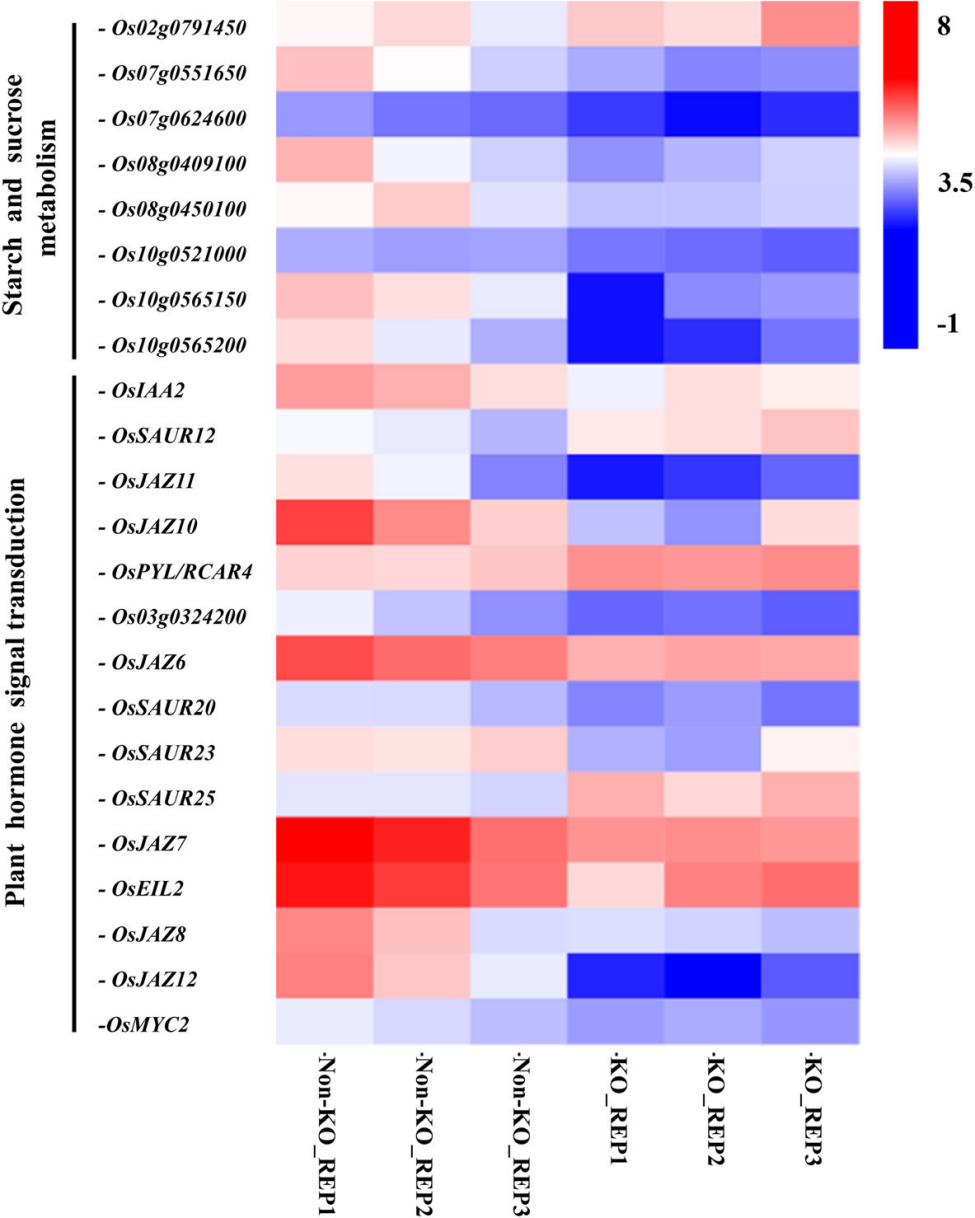
Heat map of starch/sucrose metabolism related genes and plant hormone signal transduction related genes. The red color in this figure denotes a high level of expression while blue indicates low expression. Non-KO_REP1, Non-KO_REP2, and Non-KO_REP3 denote the three control biological replicates, while KO_REP1, KO_REP2, and KO_REP3 denote the three biological replicates for the *TH1* KO line. The expression levels for each gene are shown in the heat maps using FPKM (Fragments Per Kilobase of transcript per Million fragments mapped) with a log2 scale

*TH1* has been cloned by five individual research groups using different mutants within different genetic backgrounds respectively. All these previous studies have shown that, *TH1* played an important role in lemma and palea development as well as rice fertility. Although both the amino acid change (*bsg1–2*) (Yan et al. 2013) and frame shift (*th1–1*, *bsg1–1* and *afd1*) (Li et al. 2012; Yan et al. 2013; Ren et al. 2016) mutants showed a reduction of seed setting rate, they were still partially fertile. In our study, we generated a *TH1* KO line using CRISPR/Cas9, with the target site relatively close to the translation initiation site. This difference may be responsible for the infertility of *th1*-C while all the other mutations were partially fertile with the mutant site relatively far away from the translation initiation site. Our result was also consistent with the fact that the transgenic plants of p*TH1*-*RNAi* had no filled seed (Li et al. 2012). In our RNA-seq experiment, eight genes related to starch and sucrose metabolism were differentially expressed between KO and Non-KO plants (Fig. 4), the altered expression levels of these genes might result in the defection for grain filling in the homozygous KO line.

### Plant hormone-related genes might act as downstream targets of *TH1* and involve in lemma/Palea development

**Fig. 5:**
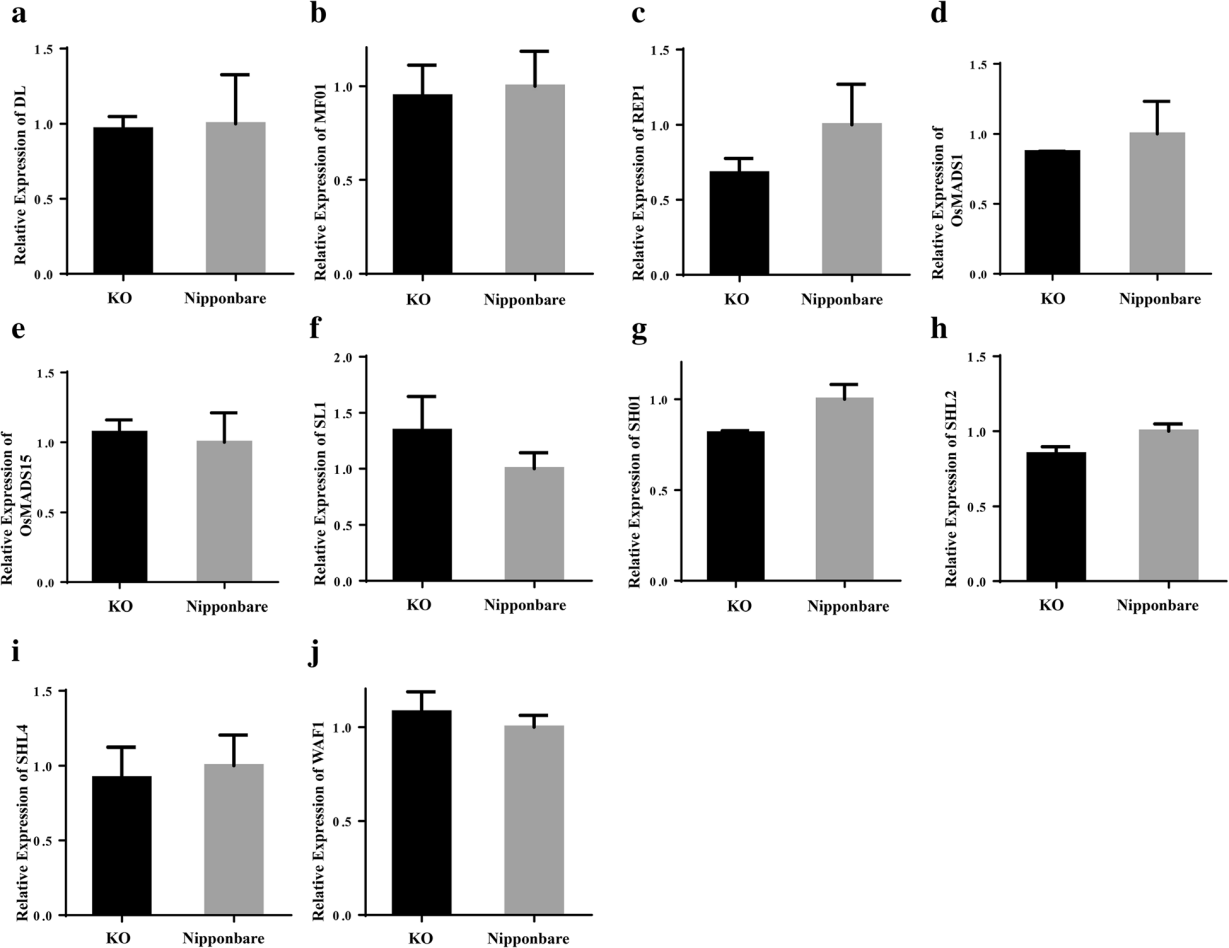
Expression levels of known lemma/palea related genes. 10 previous known lemma/palea related genes expression profiles in KO and Non-CRISPR treated Nipponbare. **a** *DL,* **b** *MFO1*/*MADS6,* **c** *REP1,* **d** *OsMADS1/LHS,* **e** *OsMADS15*, **f** *OPB*/*SL1*/*JAG*/*DH1*, **g** *SHO1*, **h** *SHL2*, **i** *SHO2*/*SHL4* and (**j**) *WAF1*. Values are shown as means ± s.d. (*n* = 3)

It has been proved that *DL*, *MFO1*/*MADS6*, *REP1*, *OsMADS1/LHS*, *OsMADS15*, *OPB*/*SL1*/*JAG*/*DH1*, *SHO1*, *SHL2*, *SHO2*/*SHL4* and *WAF1* are involved in lemma/palea development (Yamaguchi et al. 2004; Ishikawa et al. 2009; Ohmori et al. 2009; Li et al. 2010; Ohmori et al. 2009; Li et al. 2010; Luo et al. 2005; Yuan et al. 2009; Horigome et al. 2009; Xiao et al. 2009; Duan et al. 2010; Li et al. 2008; Itoh et al.; Nagasaki et al. 2007; Abe et al. 2010), however, the expression levels of these genes did not show significant difference between KO and Non-KO lines in our experiment (Additional file 7: Figure S2). We further compared the expression levels of these 10 genes between KO lines and Non-CRISPR treated Nipponbare plants, the qRT-PCR results were in accordance with the RNA-seq data (FC < 2) (Fig. 5a-j).

Some of these genes’ expression pattern within *TH1* mutant background had been studied in the previous research: In lemma and palea of *afd1*, *MFO1/OsMADS6* seemed to be close to that of wild type. In lodicules, *OsMADS1/LHS*, and *OsMADS15* transcripts were expressed in the mutants but not in wild type (Lou et al. 2016); In the *bh1* mutant, *OsMADS1/LHS*, *OsMADS15*, *REP1* and *DL* showed a down-regulation in the mutant around anthesis, however, they did not specify the developmental stage for the samples (Wei et al. 2013); The expression levels of *OsMADS1/LHS* and *MFO1/OsMADS6* were only down-regulated in 0.5-cm young panicles, but not in 8-cm young panicles for the *bsg1–1* mutant when comparing with the wild type (Yan et al. 2013); For the 1-cm young panicle, *OsMADS1/LHS*, *MFO1/OsMADS6*, *OsMADS15*, *REP1* and *DL* showed no significant difference between *bls1* and wild type (Ma et al. 2013), this is consistent with our finding from the RNA-seq data. Taken these together, it indicated that expression profile of these genes might be largely different in different genetic backgrounds, different tissues or different developmental stages.

We identified 622 DEGs related to *TH1*, and among these DEGs, a subset of genes (15 in total) related to plant hormone signal transduction was enriched (Fig. 4). Previous research has revealed that the plant hormone jasmonic acid (JA) plays an important role in determining rice spikelet morphogenesis. By repressing *OsMYC2*, a JA-inducible transcription factor would activate *OsMADS1*, an E-class gene crucial to spikelet development (Cai et al. 2014). In our experiment, *OsMYC2* showed a significant expression change. In addition, a group of jasmonate ZIM-domain protein genes showed a differential expression between the homozygous KO plants and the Non-KO plants, including *OsJAZ6*, *OsJAZ7*, *OsJAZ8*, *OsJAZ10*, *OsJAZ11* and *OsJAZ12* (Ye et al. 2009). In addition to this, five auxin-related genes (*OsIAA2*, *OsSAUR12*, *OsSAUR20*, *OsSAUR23*, *OsSAUR25*) (Song et al. 2009; Jain et al. 2006), two ethylene-related genes (*OsEIL2*, *Os03g0324200*) (Yang et al. 2015) and one ABA (abscisic acid) receptor gene (*OsPLY4*) (He et al. 2014) expressed differentially between KO and Non-KO lines. This result implied that these genes might act as downstream targets of the rice transcription factor *TH1* and be involved in rice lemma/palea development.

## Conclusions

*TH1* was characterized as a transcription factor belonging to the ALOG gene family. Loss of function in *TH1* led to defects for rice lemma and palea development as well as defects for grain filling. Our transcriptome study provided a survey for the regulatory network that controlled by *TH1*. The results indicated that hormone related genes and starch/sucrose metabolism related genes might act as downstream targets of *TH1*; they might be responsible for lemma/palea development and grain filling respectively. Most of these genes are uncharacterized or known to have other functions. Although RNA-seq provided the possibility of discovering new lemma/palea related or grain filling related genes, further research such as KO or overexpress of these genes will be needed to confirm our hypothesis.

## Methods

### Plant materials and growth conditions

Rice (*Oryza sativa* L.) plants were grown in the greenhouse at 30 °C for days and 25 °C for night. The genetic background of the all the transgenic plants was Nipponbare. For the RNA-seq experiment, the homozygous *TH1* knock out line and its control Non-KO line were derived from a T_0_ heterozygous *TH1* knock out plant: *th1*-C-15. For each progeny of *th1*-C-15, we sequenced the target site. The plants harboring two alleles of mutation were referred as homozygous KO line while plants carrying homozygous intact-*TH1* gene were used as control and referred as Non-KO line.

### Vector construction

To knock out *TH1*: Target site was designed online (http://cbi.hzau.edu.cn/crispr/), the optimal target site with low off-target score and high sgRNA score was selected. To prepare CRISPR/Cas9 binary constructs, the OsU3 sgRNA vector was used as template to generate a target-sgRNA expression cassette(s) using an overlapping PCR method, then the target-sgRNA was constructed to pYLCRISPR/Cas9-MH using a Golden Gate ligation methods. All the primers were showed in Additional file 8: Table S6.

To overexpress *TH1*: The open reading frame of *TH1* (without stop codon) was amplified from cDNA using primers Oeth1F and Oeth1R. The fragment was digested by BamHI and SmaI, and then cloned into pCAMBIA1300/35S:GFP. The primers were showed in Additional file 8: Table S6.

### Sample collection and RNA extraction

Young panicle (panicle length: ~ 2 cm) from a single plant was collected, total RNA was extracted using a Plant RNA Purification Kit: RNeasy Plant Mini Kit (74,904, QIAGEN, Germany) following the manufacturer’s instructions. RNA degradation and contamination were monitored on 1% agarose gels.

### Library construction and Illumina sequencing

Library construction and RNA-seq were performed at Beijing BioMarker Technologies (Beijing, China) in accordance with the institute’s protocols and briefly described here. RNA concentration was measured using NanoDrop 2000 (Thermo). RNA integrity was assessed using the RNA Nano 6000 Assay Kit of the Agilent Bioanalyzer 2100 system (Agilent Technologies, CA, USA). A total amount of 1 μg RNA per sample was used as input material for the RNA sample preparations. Sequencing libraries were generated using NEBNext UltraTM RNA Library Prep Kit for Illumina (NEB, USA) following manufacturer’s recommendations and index codes were added to attribute sequences to each sample. Briefly, mRNA was purified from total RNA using poly-T oligo-attached magnetic beads. Fragmentation was carried out using divalent cations under elevated temperature in NEBNext First Strand Synthesis Reaction Buffer (5X). First strand cDNA was synthesized using random hexamer primer and M-MuLV Reverse Transcriptase. Second strand cDNA synthesis was subsequently performed using DNA Polymerase I and RNaseH. Remaining overhangs were converted into blunt ends via exonuclease/polymerase activities. After adenylation of 3′ ends of DNA fragments, NEBNext Adaptor with hairpin loop structure were ligated to prepare for hybridization. In order to select cDNA fragments of preferentially 240 bp in length, the library fragments were purified with AMPure XP system (Beckman Coulter, Beverly, USA). Then 3 μl USER Enzyme (NEB, USA) was used with size-selected, adaptor-ligated cDNA at 37 °C for 15 min followed by 5 min at 95 °C before PCR. Then PCR was performed with Phusion High-Fidelity DNA polymerase, Universal PCR primers and Index (X) Primer. At last, PCR products were purified (AMPure XP system) and library quality was assessed on the Agilent Bioanalyzer 2100 system. The clustering of the index-coded samples was performed on a cBot Cluster Generation System using TruSeq PE Cluster Kit v4-cBot-HS (Illumina) according to the manufacturer’s instructions. After cluster generation, the library preparations were sequenced on Illumina Hiseq Xten PE150 platform (paired end 150 bp) and paired-end reads were generated. All the raw data have been submitted to the NCBI SRA database under accession number of PRJNA470824.

### RNA-seq data analysis

Raw data (raw reads) of fastq format were firstly processed through in-house perl scripts. In this step, clean data (clean reads) were obtained by removing reads containing adapter, reads containing ploy-N and low-quality reads from raw data. At the same time, Q20, Q30, GC-content and sequence duplication level of the clean data were calculated. These clean reads were then mapped to the reference genome sequence (Os-Nipponbare-Reference-IRGSP-1.0: http://rapdb.dna.affrc.go.jp/download/irgsp1.html). Only reads with a perfect match or one mismatch were further analyzed and annotated based on the reference genome. Tophat2 tools soft were used to map with reference genome (Kim et al. 2013). Gene function was annotated based on the following databases: Nr (NCBI non-redundant protein sequences); Nt (NCBI non-redundant nucleotide sequences); Pfam (Protein family); KOG/COG (Clusters of Orthologous Groups of proteins); Swiss-Prot (A manually annotated and reviewed protein sequence database); KO (KEGG Ortholog database); GO (Gene Ontology). Differential expression analysis of two groups (KO samples and Non-KO samples) was performed using the edgeR package. edgeR is designed for the analysis of replicated count-based expression data. In principle, edgeR will combine three KO lines and compared to the combination of three Non-KO lines. Genes with FDR (false discovery rate) < 0.05 & FC (Fold Change) were assigned as differentially expressed.

### Gene expression, KEGG pathway enrichment analysis

KEGG pathway enrichment analysis (http://www.genome.jp/kegg/) was performed to identify differentially expressed genes (DEGs). KOBAS (Mao et al. 2005) software was used to test the statistical enrichment of differential expression genes in KEGG pathways (E ≤ 1e-5). Gene expression levels were estimated by FPKM values (Fragments Per Kilobase of transcript per Million fragments mapped).

### qRT-PCR

First-strand cDNA was synthesized from 1 μg total RNA using the PrimeScript™ RT reagent Kit (with gDNA Eraser) (RR047A, Takara, Japan) according to the manufacturer’s instructions. qRT-PCR were carried out using the ChamQTM SYBR® qPCR Master Mix (Q311–01, Vazyme, China) on the LightCycler® 480II (Roche) according to the manufacturer’s instructions. Rice Ubiquitin (*Os03g0234200*) was used as internal reference and gene expression level was normalized to the Ubiquitin expression level. The primers were showed in Additional file 9: Table S7.

### Pollen viability test

Pollen viability was estimated by IKI (iodine potassium iodide) straining. The mature pollen grains were collected from at least three unopened flowers randomly for WT and *TH1* homozygous knock out plants, respectively. Then put them on a microscope slide immediately, dripped a drop of the IKI solution on the pollen and covered with a coverslip. Pollen viability counts were made 5 min after pollen was placed on an IKI solution. Pollen grains stained dark (dark red or brown color) were counted as alive.

## Additional files

Additional file 1: **Table S1.** The target site sequence of the 20 T_0_ transgenic plants. (DOCX 20 kb)

Additional file 2: **Table S2.** All genes expression profile. (XLSX 3158 kb)

Additional file 3: **Table S3.** Differentially expressed genes. (XLSX 111 kb)

Additional file 4: **Table S4.** Genes assigned to KEGG. (XLS 50 kb)

Additional file 5: **Table S5.** Genes assigned to KEGG pathway. (XLSX 15 kb)

Additional file 6: **Figure S1.** Validation of RNA-seq by qPCR. (TIF 685 kb)

Additional file 7: **Figure S2.** Heat map of previously known as lemma/palea-related genes. (JPG 866 kb)

Additional file 8: **Table S6.** Primers used for constructing the transgenic line. (DOCX 16 kb)

Additional file 9: **Table S7.** Primers used for qPCR for this study. (DOCX 18 kb)

## Abbreviations

ABA: Abscisic Acid
DEGs: Differentially Expressed Genes
FC: Fold Change
FDR: False Discovery Rate
FPKM: Fragments Per Kilobase of transcript per Million fragments mapped
GFP: Green Fluorescent Protein
JA: Jasmonic Acid
KO: Knock Out
qRT-PCR: Quantitative Reverse Transcription Polymerase Chain Reaction
WT: Wild Type
