# Supplementary material for: Analysing the rice young panicle transcriptome reveals the gene regulatory network controlled by TRIANGULAR HULL1
Source: Rice (N Y). 2019 Feb 6;12:6. doi: 10.1186/s12284-019-0265-2 (PMC6890884; doi:10.1186/s12284-019-0265-2)
Supplement: Supplementary file 1 — Table S1. The target site sequence of the 20 T0 transgenic plants. (DOCX 20 kb) [file 12284_2019_265_MOESM1_ESM.docx]

**Table S1. The target site sequence of the 20 T_0_ transgenic plants**

| Wild-type | accATCACATGATGTCGGGC - GGCgggcaagacc |  |
| --- | --- | --- |
|  |  |  |
| *th1*-C-1 | accATCACATGATGTCGGGC **G** GGCgggcaagacc | +1 |
|  | accATCACATGATGTCGGGC **T** GGCgggcaagacc | +1 |
| *th1*-C-2 | accATCACATGATGTCGGGC **T** GGCgggcaagacc | +1 |
|  | accATCACATGATGTCGGGC **A** GGCgggcaagacc | +1 |
| *th1*-C-3 | accATCACATGATGTCGGGC **T** GGCgggcaagacc | +1 |
|  | accATCACATGATGTCGGGC **G** GGCgggcaagacc | +1 |
| *th1*-C-4 | accATCACATGATGTCGGGC - **-** **-** **-** gggcaagacc | -3 |
|  | accATCACATGATGTCGGGC - **-** **-** **-** **-** **-** **-** **-** aagacc | -7 |
| *th1*-C-5 | accATCACATGATGTCGGGC - GGCgggcaagacc | 0 |
|  | accATCACATGATGTCGGGC - GGCgggcaagacc | 0 |
| *th1*-C-6 | accATCACATGATGTCGGGC **T** GGCgggcaagacc | +1 |
|  | accATCACATGATGTCGGGC **T** GGCgggcaagacc | +1 |
| *th1*-C-7 | accATCACATGATGTCGGGC **T** GGCgggcaagacc | +1 |
|  | accATCACATGATGTCGGGC **A** GGCgggcaagacc | +1 |
| *th1*-C-8 | accATCACATGATGTCGGGC **T** GGCgggcaagacc | +1 |
|  | accATCACATGATGTCGGGC **T** GGCgggcaagacc | +1 |
| *th1*-C-9 | accATCACATGATGTCGGGC - GGCgggcaagacc | 0 |
|  | accATCACATGATGTCGGGC - GGCgggcaagacc | 0 |
| *th1*-C-12 | accATCACATGATGTCGGGC - GGCgggcaagacc | 0 |
|  | accATCACATGATGTCGGGC - **-** **-** **-** gggcaagacc | -3 |
| *th1*-C-13 | accATCACATGATGTCGGGC - GGCgggcaagacc | 0 |
|  | accATCACATGATGTCGGGC **T** GGCgggcaagacc | +1 |
| *th1*-C-14 | accATCACATGATGTCGGGC **T** GGCgggcaagacc | +1 |
|  | accATCACATGATGTCGGGC **A** GGCgggcaagacc | +1 |
| *th1*-C-15 | accATCACATGATGTCGGGC - GGCgggcaagacc | 0 |
|  | accATCACATGATGTCGGG **-** - GGCgggcaagacc | -1 |
| *th1*-C-16 | accATCACATGATGTCGGGC - GGCgggcaagacc | 0 |
|  | accATCACATGATGTCGGGC - GGCgggcaagacc | 0 |
| *th1*-C-17 | accATCACATGATGTCGGGC - GGCgggcaagacc | 0 |
|  | accATCACATGATGTCGGGC - GGCgggcaagacc | 0 |
| *th1*-C-19 | accATCACATGATGTCGGGC **G** GGCgggcaagacc | +1 |
|  | accATCACATGATGTCGGGC **A** GGCgggcaagacc | +1 |
| *th1*-C-20 | accATCACATGATGTCGGGC **T** GGCgggcaagacc | +1 |
|  | accATCACATGATGTCGGGC **T** GGCgggcaagacc | +1 |
| *th1*-C-22 | accATCACATGATGTCGGGC **T** GGCgggcaagacc | +1 |
|  | accATCACATGATGTCGGGC **T** GGCgggcaagacc | +1 |
| *th1*-C-23 | accATCACATGATGTCGGGC **T** GGCgggcaagacc | +1 |
|  | accATCACATGATGTCGGGC **A** GGCgggcaagacc | +1 |
| *th1*-C-24 | accATCACATGATGTCGGGC - **- -** **-** gggcaagacc | -3 |
|  | accATCACATGATGTCGGGC - **-** **-** **-** gggcaagacc | -3 |

The sequence of the target sites for two alleles of each transgenic plant. The mutant site are shown in bold, and the protospacer adjacent motif (PAM) site is underlined. The numeric value on the right represents the mutant type for each allele, + represents nucleotide insertion, - represents nucleotide deletion, the number represents the insertion or deletion number of nucleotide.
