# Supplementary figures and images for: Analysing the rice young panicle transcriptome reveals the gene regulatory network controlled by TRIANGULAR HULL1
Source: Rice (N Y). 2019 Feb 6;12:6. doi: 10.1186/s12284-019-0265-2 (PMC6890884; doi:10.1186/s12284-019-0265-2)

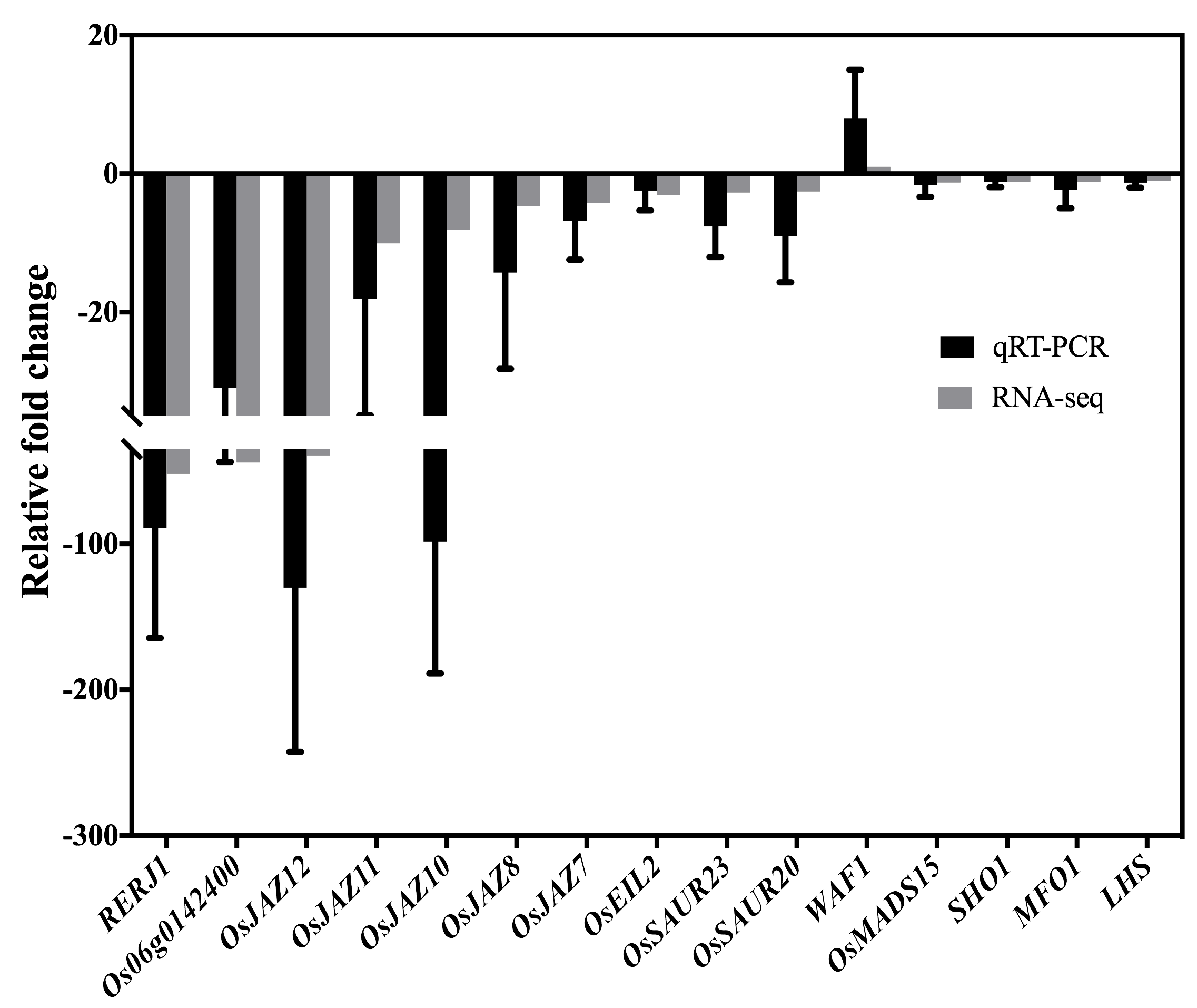

Supplement: Supplementary file 6 — Figure S1. Validation of RNA-seq by qPCR. (TIF 685 kb) [file 12284_2019_265_MOESM6_ESM.tif]

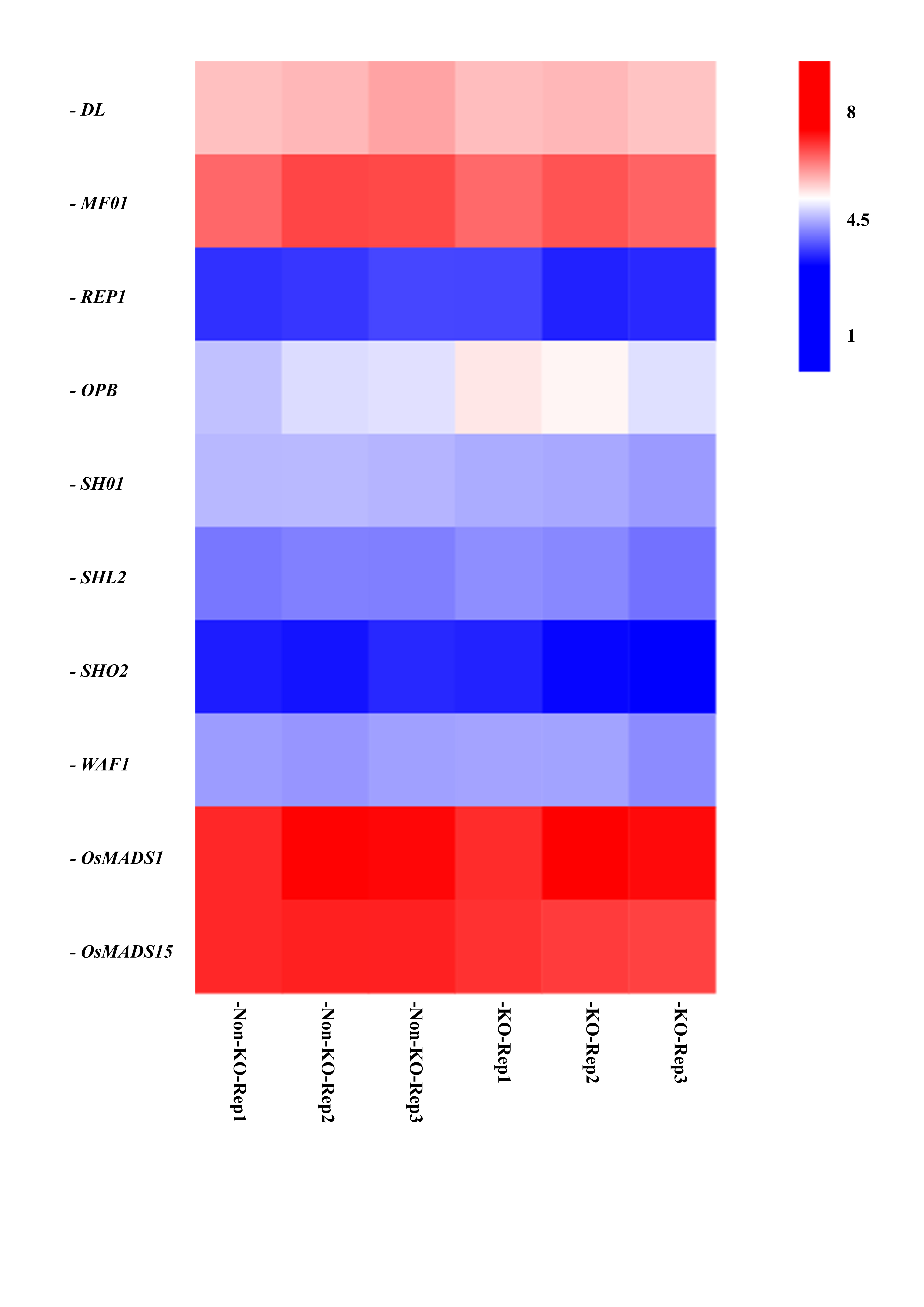

Supplement: Supplementary file 7 — Figure S2. Heat map of previously known as lemma/palea-related genes. (JPG 866 kb) [file 12284_2019_265_MOESM7_ESM.jpg]
