# Supplementary material for: Analysing the rice young panicle transcriptome reveals the gene regulatory network controlled by TRIANGULAR HULL1
Source: Rice (N Y). 2019 Feb 6;12:6. doi: 10.1186/s12284-019-0265-2 (PMC6890884; doi:10.1186/s12284-019-0265-2)
Supplement: Supplementary file 8 — Table S6. Primers used for constructing the transgenic line. (DOCX 16 kb) [file 12284_2019_265_MOESM8_ESM.docx]

| Application | Primers | Sequence (5’→3’) |
| --- | --- | --- |
| 1^st^ PCR | U-F | CTCCGTTTTACCTGTGGAATCG |
|  | gR-R | CGGAGGAAAATTCCATCCAC |
|  | gRT-TH1 | TCACATGATGTCGGGCGGCgttttagagctagaaat |
|  | OsU3T-TH1 | GCCGCCCGACATCATGTGATgccacggatcatctgc |
| 2^nd^ PCR |  |  |
| Site B-L | Pps-GGL | TTCAGAggtctcTctcgACTAGTATGGAATCGGCAGCAAAGG |
| Site B-R | Pgs-GGR | AGCGTGggtctcGaccgACGCGTATCCATCCACTCCAAGCTC |
| Flanking Primers | PB-L | GCGCGCgGTctcGCTCGACTAGTATGG |
|  | PB-R | GCGCGCggtctcTACCGACGCGTATCC |
| Identification for the target site | TH1-F | ATCGGCTGCTACCTGACC |
|  | TH1-R | CGAACGTGTTCCAGTCCC |
| Overexpression of *TH1* | TH1-OE-F | cgggatccATGGATCGTCACCATCACC |
|  | TH1-OE-R | tcccccgggCGGGATGATGAACTGCG |

**Table S6. Primers used for constructing the transgenic line**

For gRT-TH1 and OsU3T-TH1, the lower case letters in the primer indicated the adaptor using for the construction system.

For Pps-GGL and Pgs-GGR, the lower case letters “ggtctc” in the primer indicated the *BsaI* site. The “ctcg” in Pps-GGL and “accg” in Pgs-GGR indicated the *BsaI*-cutting non-palindromic end that compactible for ligating to pYLCRISPR/Cas9-MH.

For TH1-OE-F and TH1-OE-R, the lower case letters in the primer indicated the adaptor with BamHI and SmaI respectively, which used for the cloning the *TH1* into pCAMBIA1300/35S:GFP.
