# Supplementary material for: Analysing the rice young panicle transcriptome reveals the gene regulatory network controlled by TRIANGULAR HULL1
Source: Rice (N Y). 2019 Feb 6;12:6. doi: 10.1186/s12284-019-0265-2 (PMC6890884; doi:10.1186/s12284-019-0265-2)
Supplement: Supplementary file 9 — Table S7. Primers used for qPCR for this study. (DOCX 18 kb) [file 12284_2019_265_MOESM9_ESM.docx]

Table S7. Primers used for qPCR for this study

| Gene ID | Gene name | Sequence(5'->3') |
| --- | --- | --- |
| Os03g0180900 | *OsJAZ11* | Forward primer: GAAAGACACGCAAGAAAGGAAG |
|  |  | Reverse primer: GAAATCGTCGAACACCACCA |
| Os03g0181100 | *OsJAZ10* | Forward primer: GGCTTTGACCAAAAACGACTTA |
|  |  | Reverse primer: GTAAAAACTCTACGTCGCCATG |
| Os04g0608300 | *OsSAUR20* | Forward primer: TCGAGAAGAACAAGAAGGACAA |
|  |  | Reverse primer: TACTACGTTCCTCTTCATCTGC |
| Os04g0662400 | *OsSAUR23* | Forward primer: TGCTTCAAGGGATGGACAG |
|  |  | Reverse primer: CTTTTGCTTATCGGTGGCTTAG |
| Os07g0615200 | *OsJAZ7* | Forward primer: GCTGTTACAGACAACACCAAG |
|  |  | Reverse primer: GCAATAGGCATATCAGAAGCAT |
| Os07g0685700 | *OsEIL2* | Forward primer: TAAGGGCATAGAGAGAGTCAGA |
|  |  | Reverse primer: GATCGTAGCACAGACTGACTTA |
| Os09g0439200 | *OsJAZ8* | Forward primer: GATCAGCAAAGCGACGAAAG |
|  |  | Reverse primer: GGGAAGTTCTCAAAGACGACTA |
| Os10g0392400 | *OsJAZ12* | Forward primer: AACGAGCGAACCATACAAGAAG |
|  |  | Reverse primer: AGATAAATCTTCAGAGCCCGAG |
| Os02g0682200 | *MFO1* | Forward primer: GAAAGGTACCAACATTGTTGCT |
|  |  | Reverse primer: CGCTGCAAAGCTTCAAATTTTG |
| Os04g0509300 | *SHO1* | Forward primer: AACACTTCAAGGAAAGGTTTCG |
|  |  | Reverse primer: CGCAGGTTGAAGATCTGTTTAG |
| Os07g0164000 | *WAF1* | Forward primer: GCTTCCTTCATTTCTGGTCAAA |
|  |  | Reverse primer: GTATGAAGCTCTGAGTCTCTCC |
| Os03g0215400 | *LHS* | Forward primer: GTCAAGAAAGAACCAAGCACTG |
|  |  | Reverse primer: GCACTGGTTTCCTGTAACTTTT |
| Os07g0108900 | *OsMADS15* | Forward primer: AGGCAAAGATTGAGACCATACA |
|  |  | Reverse primer: GGAGTTCTTTGAGATTCAGGGA |
| Os04g0301500 | *RERJ1* | Forward primer: GGATAGTGTCCAAATGAAGCAG |
|  |  | Reverse primer: CCAAAACGCATGACTCCATATA |
| Os06g0142400 |  | Forward primer: TTCGCTTCAGACTTTCTCTTCA |
|  |  | Reverse primer: CTTAACCCCTGCAATGTTCG |
| Os03g0299700 |  | Forward primer: GCCCAAGTGTTGTTGCTCTG |
|  |  | Reverse primer: GCACGGAGCAGTAGTACGAA |
| Os06g0598850 |  | Forward primer: GTGTCGAATTGCCTGTTCCG |
|  |  | Reverse primer: CTCATACTTGGACCTCCGGC |
| Os11g0157000 |  | Forward primer: GTGGAAGCAAGGGTGGATGA |
|  |  | Reverse primer: AAGTACCCGGCCATCGTTTC |
| Os11g0674400 |  | Forward primer: CCTCTCTGGTGCCCTTTCAAT |
|  |  | Reverse primer: GCTCTCTCACTTGGGGAAGG |
| Os03g0215200 | *DL* | Forward primer: GACATCAAATGAGGGTAGCCC |
|  |  | Reverse primer: GCAGCTTTGATACGCTGTATTT |
| Os09g0410500 | *REP1* | Forward primer: TCATCATCACCTGATCACTAGC |
|  |  | Reverse primer: CTTCTTCTTCCTCTCGATCTCC |
| Os01g0129200 | *SL1* | Forward primer: AAGCATCCAGGGTTACTAAG |
|  |  | Reverse primer: CGTCGTCGTGTAGAGGTAGT |
| Os01g0527600 | *SHL2* | Forward primer: GCAAAGAATCCCTGTCTTCATC |
|  |  | Reverse primer: ACACCAAACAATCAACCAGATG |
| Os03g0449200 | *SHL4* | Forward primer: CCACCGCATGCCATTACCG |
|  |  | Reverse primer: ACAGTAAACTTCTCATGGGTGTC |
| Os02g0811000 | *TH1* | Forward primer: TCGCCCGGATCCACGGACACCTCTC |
|  |  | Reverse primer: ATCTCCTCCTCCACTGCTCCACTCG |
| Os03g0234200 | *Ubiquitin* | Forward primer: CTGTCAACTGCCGCAAGAAG |
|  |  | Reverse primer: GGCGAGTGACGCTCTAGTTC |
